# Mapping of pediatric allergy structures in Italy: a nationwide survey

**DOI:** 10.1186/s13052-026-02261-2

**Published:** 2026-04-22

**Authors:** Giulio Dinardo, Mattia Giovannini, Francesca Galletta, Angela Klain, Maria de Filippo, Cristiana Indolfi, Gian Luigi Marseglia, Michele Miraglia del Giudice, Sara Manti

**Affiliations:** 1 Department of Woman, Child and General and Specialized Surgery, University of Campania “Luigi Vanvitelli”, Naples, Italy; 2 Allergy Unit, Meyer Children’s Hospital IRCCS, Florence, Italy; 3 Department of Health Sciences, University of Florence, Florence, Italy; 4 Pediatric Unit, Department of Human Pathology in Adult and Developmental Age “Gaetano Barresi”, University of Messina, 98124 Messina, Italy; 5 Pediatric Unit, Department of Clinical, Surgical, Diagnostic and Pediatric Sciences, University of Pavia, Pavia, Italy; 6 Department of Maternal Infantile and Urological Sciences, AOU Policlinico Umberto I, Rome, Italy; 7 Department of Clinical, Surgical, Diagnostic and Pediatric Sciences, University of Pavia, Pavia, Italy; 8 Pediatric Clinic, Fondazione IRCCS Policlinico San Matteo, Pavia, Italy

**Keywords:** Pediatric allergy, Immunology, Health services accessibility, Surveys and questionnaires, Italy, Asthma, Atopic diseases, Health care disparities, Delivery of health care, Telemedicine

## Abstract

**Background:**

Pediatric allergology and immunology has expanded markedly over recent decades, paralleling the rising prevalence of allergic diseases. National data on the availability and organization of dedicated services in Italy are lacking. This project aimed to provide the first nationwide mapping of Italian Society of Pediatric Allergy and Immunology (SIAIP)-affiliated pediatric allergology and immunology structures, describing their geographical distribution and main organizational features of service delivery.

**Methods:**

Between 2023 and 2025, a structured nationwide mapping was conducted by regional SIAIP representatives. Data referred to the years 2024–2025 and included geographic location, weekly clinical activity, patient contact modalities (telephone and e-mail), and availability of digital services such as telemedicine. Data were summarized using descriptive statistics and reported as frequencies and percentages.

**Results:**

A total of 188 pediatric allergology and immunology territorial structures were identified across Italy. Their distribution was uneven: 86 were located in Northern Italy (45.7%), 42 in Central Italy (22.3%), and 60 in Southern Italy and the Islands (31.9%), highlighting marked regional heterogeneity. Clinical activity was frequently limited, with many structures operating only one to two days per week. Communication pathways were variable and often restricted to scheduled hours or mediated by booking services. Direct clinician contact was inconsistently available. Telemedicine and digital follow-up services were rarely implemented, indicating limited integration of remote care in routine practice.

**Conclusions:**

The Italian pediatric allergology network shows important structural limitations, including regional disparities, reduced service availability, and minimal integration of telemedicine. These findings provide a framework for healthcare planning and underline the urgency of strengthening workforce organization, service capacity, and digital health implementation.

**Supplementary Information:**

The online version contains supplementary material available at 10.1186/s13052-026-02261-2.

## Background

Over the past decades, pediatric healthcare has progressively shifted toward specialized and multidisciplinary models of care, driven by the increasing complexity of childhood diseases, longer survival of patients with chronic conditions, and advances in diagnostic and therapeutic pathways [1]. In this context, pediatric subspecialties play a key role in ensuring appropriate management for children with complex, severe, or rare disorders. Nevertheless, the organization of subspecialty services remains challenged by workforce limitations, geographic disparities in specialist availability, and incomplete integration with primary care, ultimately impacting access and continuity of care [2]. Within this evolving landscape, pediatric allergology and immunology has gained increasing clinical relevance due to the worldwide rise in allergic and immune-mediated diseases in childhood. These conditions often require structured diagnostic work-up, long-term follow-up, and multidisciplinary collaboration, as well as the ability to manage acute and potentially life-threatening manifestations, such as severe asthma exacerbations, food allergy reactions, and immune dysregulation disorders [1, 2]. Despite this growing demand, national-level information describing the distribution, organizational models, and accessibility of pediatric allergology and immunology services in Italy is currently lacking. This gap limits the possibility of identifying underserved areas, supporting workforce planning, and promoting standardized models of care [3]. To address this unmet need, the Italian Society of Pediatric Allergy and Immunology (SIAIP) promoted a nationwide mapping initiative aimed at identifying and characterizing SIAIP-affiliated pediatric allergology and immunology territorial structures across Italy. The primary objectives were to describe their geographic distribution and organizational setting, assess service availability and accessibility, and identify potential gaps and priorities to support a more equitable and coordinated pediatric allergy care network nationwide.

## Materials and methods

A nationwide mapping project was conducted between 2023 and 2025 to identify and characterize pediatric allergology and immunology territorial structures across Italy. The initiative targeted members of the Italian Society of Pediatric Allergy and Immunology (SIAIP) and pediatricians actively involved in pediatric allergology and immunology services. Data collection was coordinated by the SIAIP network through junior and senior regional representatives, who gathered information on structures operating within their respective regions. A structured questionnaire was developed by the coordinating team of the Mapping Project to obtain a standardized overview of pediatric allergology services nationwide. The questionnaire was distributed through the SIAIP regional representatives, who forwarded it to the directors or responsible physicians of the pediatric allergology and immunology structures within their region. Each structure designated a single representative responsible for completing the data collection form and returning it through the regional representatives, ensuring standardized reporting and minimizing the risk of duplicate entries. When necessary, regional representatives contacted the centers to clarify incomplete or unclear information. Data were collected for each structure and referred to the years 2024–2025. The questionnaire included 16 items organized into four thematic sections and collected information on: (i) identification of the structure (official name, type, and website, when available); (ii) medical coordination (name of the director/coordinator and contact details); (iii) modalities of access (booking procedures and practical information for families); and (iv) organization of clinical activity and available communication tools. The complete English version of the structured questionnaire is provided as Supplementary Material 1 (Supplement 1).

### Statistical analysis

The analysis was descriptive. Categorical variables were summarized as absolute frequencies and percentages. Regional distribution was reported overall and according to macro-areas (Northern, Central, and Southern Italy and Islands). No imputation was performed, as the mapping achieved complete coverage with no missing data. All analyses were performed using IBM SPSS Statistics (IBM Corp., Armonk, NY, USA).

## Results

### Pediatric allergology structures

**Fig. 1:**
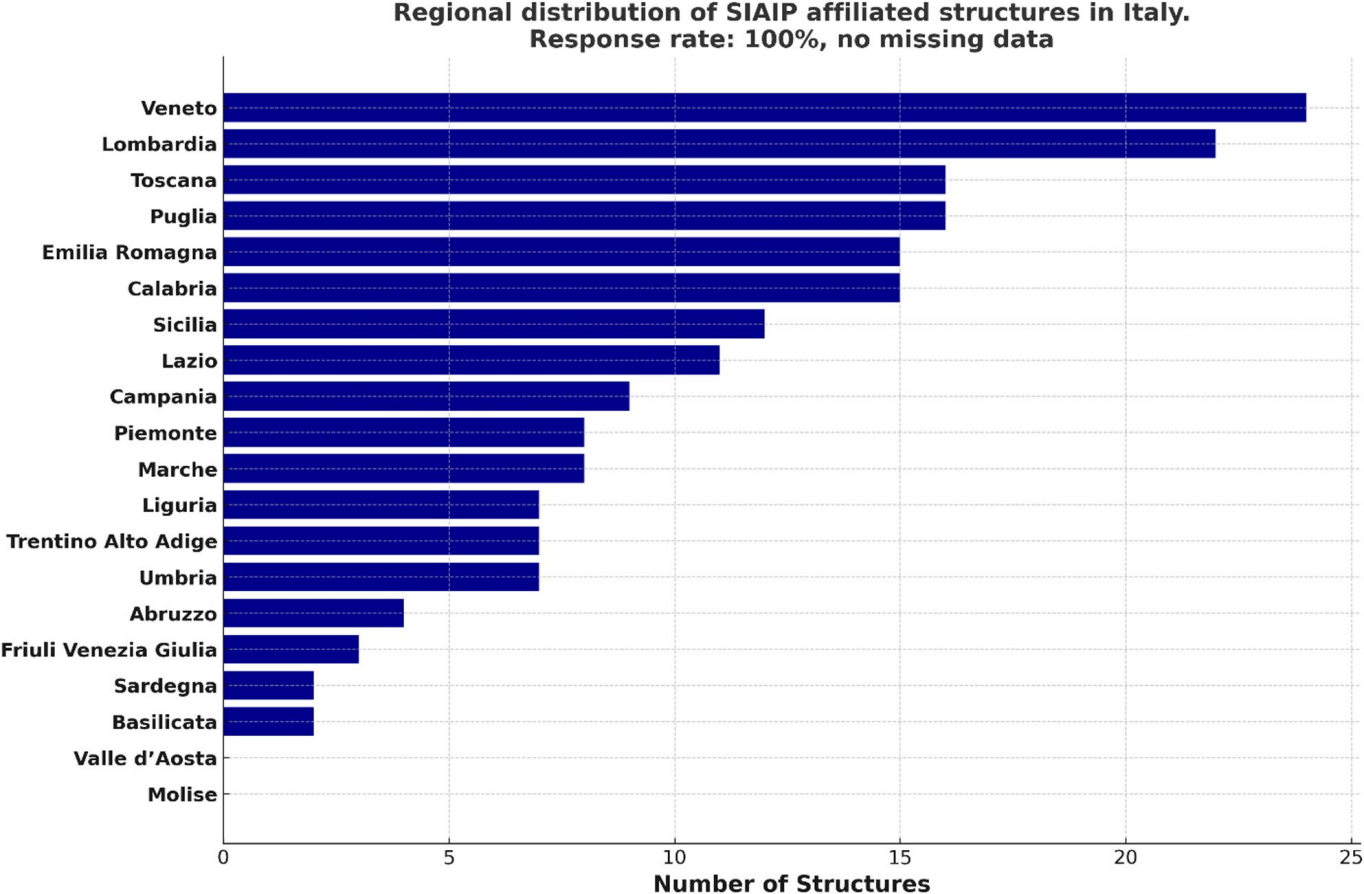
Regional distribution of SIAIP-affiliated pediatric allergy and immunology territorial structures in Italy (*n* = 188)

A total of 188 pediatric allergy and immunology structures completed the survey, and their geographic distribution is illustrated in Fig. 1. Of these, 147 (78.2%) were hospital-based, 31 (16.5%) were academic structures embedded within teaching hospitals, and 10 (5.3%) were IRCCS research institutions affiliated with hospitals. The regional representation was not uniform and did not consistently align with the population density or healthcare infrastructure. Most territorial structures were concentrated in Northern Italy (86 structures 45.7%), reflecting the region’s historically greater investment in specialized pediatric services. In contrast, Central Italy accounted for 42 structures (22.3%), and Southern Italy and the Islands hosted 60 structures (31.9%), often dispersed across larger geographic areas with fewer tertiary care hubs. Notably, while most Italian regions had at least one pediatric allergy structure, Basilicata and Sardegna reported only two structures each, raising concerns about local service availability and travel burden for families. Furthermore, Molise and Valle d’Aosta, two of the country’s smallest and most peripheral regions, had no structures at all. This uneven geographic distribution underscores potential disparities in access to care, particularly in rural or underserved areas, and highlights the need for national strategies to support equitable service provision across regions of the country.

### Organizational structure

**Fig. 2:**
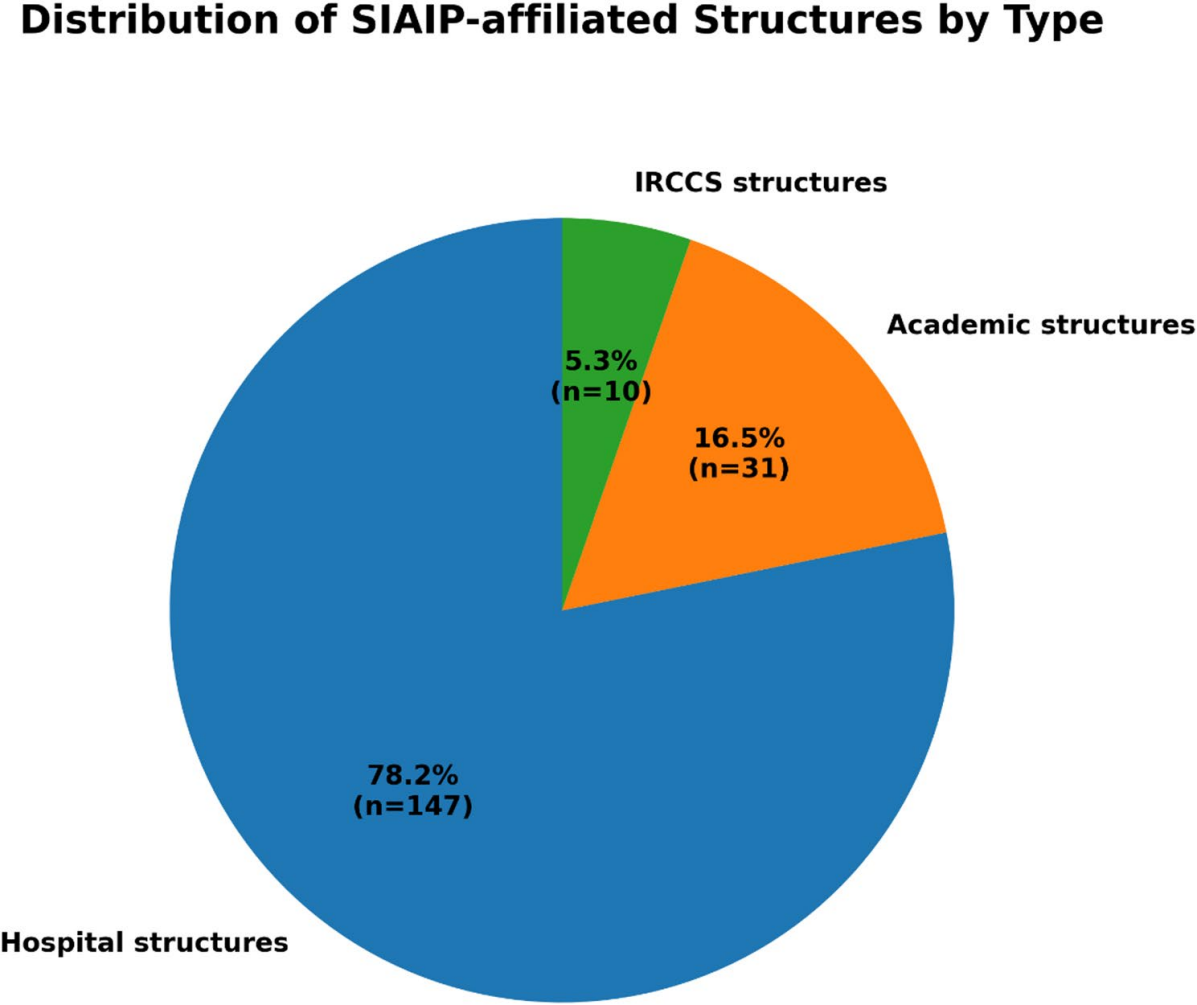
Distribution of SIAIP-affiliated pediatric allergy and immunology territorial structures by structure category

The study encompassed 188 pediatric allergy and immunology structures, the majority being hospital-based (*n* = 147, 78.2%), followed by academic structures within teaching hospitals (*n* = 31, 16.5%) and IRCCS research institutions affiliated with hospitals (*n* = 10, 5.3%) **(**Fig. 2**)**. The organizational models varied across institutions. Overall, 41 centers (21.8%) were embedded within pediatric or multidisciplinary units, 53 centers (28.2%) were subdivisions of broader hospital departments, and 18 centers (9.6%) operated as fully independent units with dedicated administrative and clinical autonomy. Among the 147 hospital-based centers, most were integrated within existing pediatric or general departments: 48 centers (32.7%) functioned as outpatient pediatric clinics, 38 (25.9%) as divisions within established units, and 49 (33.3%) as departmental subdivisions. Only 12 hospital centers (8.2%) operated as autonomous units formally dedicated to pediatric allergy and immunology. In contrast, academic centers demonstrated a higher degree of structural independence. Of the 31 university-affiliated centers, 18 (58.1%) were fully independent units, while 13 centers remained integrated within pediatric units or larger hospital departments.

### Clinical activity and patient communication

**Fig. 3:**
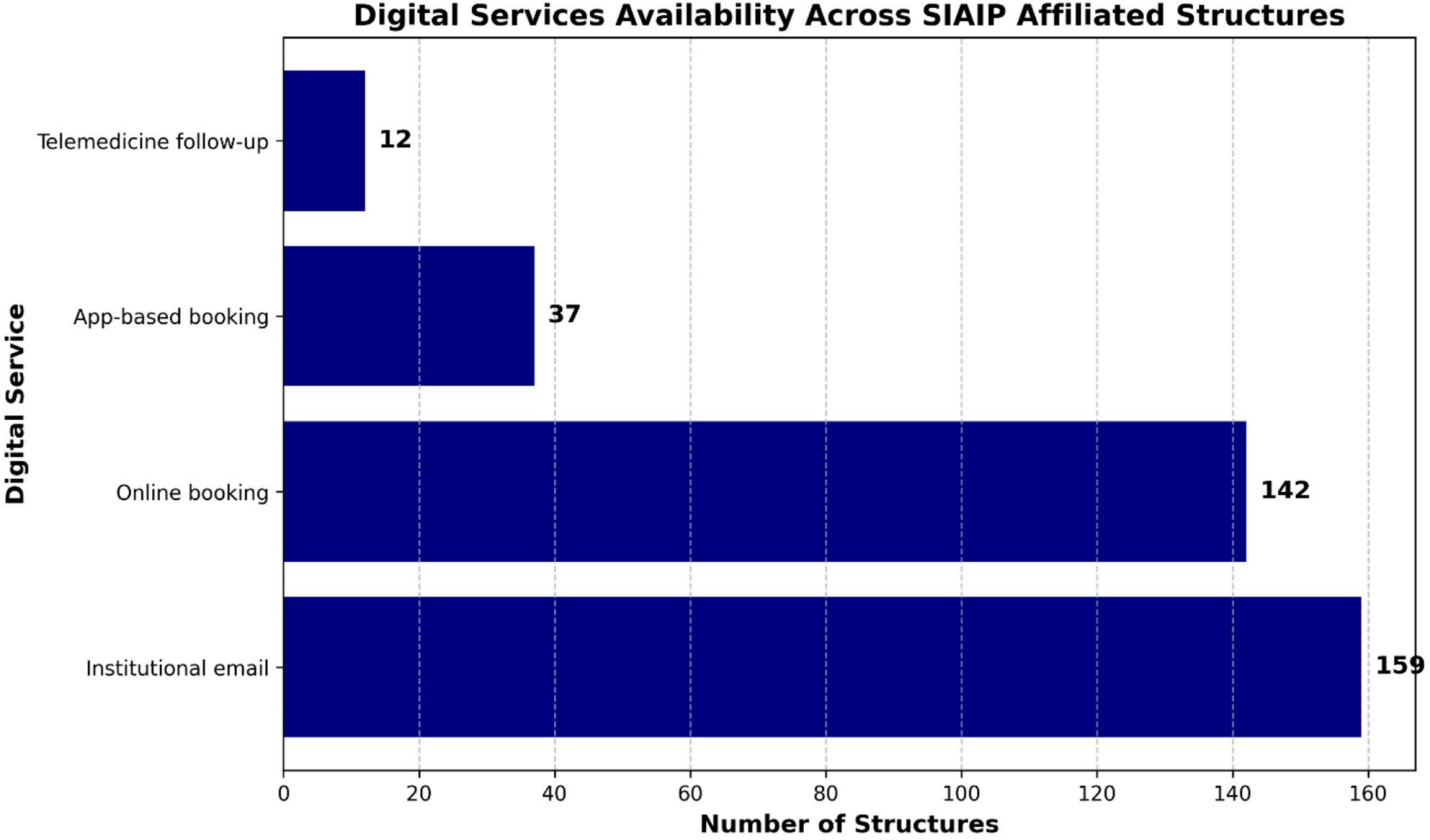
Availability of digital services in SIAIP-affiliated pediatric allergy and immunology territorial structures

The frequency and intensity of clinical activity varied substantially across 188 structures. Ninety-six structures (51.1%) reported conducting outpatient services 1–2 days per week, reflecting a part-time operational model often constrained by limited staff and shared resources. An additional 32 structures (17.0%) operated less than once a week, primarily in smaller facilities or regions with limited specialist availability. At the other end of the spectrum, 60 structures (31.9%) maintained a full clinical schedule of six days per week, typically associated with larger hospital units or academic and research institutions with high patient volumes and broader service scopes. Regarding patient access to clinical staff outside the visit hours, considerable variation was observed. Forty (21.3%) structures did not provide telephone services, limiting direct communication between families and healthcare providers. Seventy-nine structures (42.0%) offered a scheduled landline telephone service, usually confined to specific days or time slots only. Thirty-eight structures (20.2%) ensured medical availability via landline or mobile phone five days a week, allowing for more consistent patient-provider interaction. Remarkably, 31 structures (16.5%) provided unofficial 24/7 telephone availability, either through personal mobile numbers or institutional on-call systems, highlighting a high level of commitment, albeit outside the formal service arrangements. In terms of digital communication, Digital communication was more widespread. A total of 159 structures (84.6%) offered a dedicated institutional email address for patient interaction, which was used to request information, transmit documentation, or manage appointments. This channel is also commonly used, e.g., to request general information, forward clinical documentation, manage administrative procedures, or receive medical advice. Additionally, 142 structures (75.5%) supported online booking systems via hospital or regional platforms, and 37 of these (19.7%) also offered mobile app-based access, with higher adoption in regions such as Calabria, Toscana, and Lombardia. However, 46 structures (24.5%) continued to rely exclusively on telephone scheduling **(**Fig. 3**).**

Despite the growing interest in telemedicine, only 12 structures (6.4%) reported offering telehealth follow-up consultations, a service primarily available in IRCCS or academic structures with dedicated infrastructure. This suggests that while digital tools for booking and communication are widespread, virtual clinical services remain underdeveloped, potentially limiting care continuity for patients in remote or underserved regions.

## Discussion

This national mapping identified 188 SIAIP-affiliated pediatric allergology and immunology territorial structures across Italy, revealing substantial geographic and organizational heterogeneity. Nearly half of the structures were located in Northern Italy, while availability in Southern regions and the Islands remained uneven. In addition, outpatient activity was frequently limited to one or two days per week and digital services, including telemedicine, were rarely implemented [4]. Collectively, these findings suggest that access to pediatric allergology services is not uniform across the country and may depend on both regional healthcare organization and local workforce distribution. Comparable access disparities have been reported internationally. Pongdee et al. described major obstacles for rural populations in the United States, including low specialist density, limited infrastructure, and higher rates of undertreatment of common allergic conditions such as asthma and food allergy [5]. Such barriers can translate into delayed diagnosis and suboptimal disease control, ultimately worsening patient outcomes and increasing morbidity [5]. Although the Italian healthcare system differs from the US model, the parallel highlights a shared challenge: ensuring timely access to specialist care in geographically underserved settings. Beyond geographic distribution, organizational variability emerged as a key feature. Most territorial structures operated as integrated outpatient services within larger pediatric or multidisciplinary departments, whereas only a minority functioned as fully autonomous units with dedicated administrative and clinical recognition. This fragmentation may influence service capacity, continuity of care, and the ability to develop structured training and research programs. Similar issues have been reported in other Italian pediatric subspecialties, such as pediatric endocrinology, where services often remain embedded within general pediatric units with limited autonomy [5]. In our mapping, the restricted weekly availability observed in many structures further supports this interpretation, as limited clinic days may contribute to longer waiting lists and difficulties in ensuring appropriate follow-up for chronic allergic diseases. Digital accessibility and communication tools represented another relevant aspect. Although families increasingly request flexible models of care, telemedicine adoption remained limited in the mapped structures [4]. Experience gained during the COVID-19 pandemic, together with recommendations such as those reported in the EAACI-ARIA position paper [6], suggest that telemedicine can reduce geographical barriers, support follow-up of chronic diseases, and improve access for patients living far from referral facilities. Pongdee et al. also emphasized the potential of digital health technologies to enhance continuity of care and reduce inequities. However, the effective implementation of telemedicine requires more than the availability of technological tools; it also depends on staff training, shared organizational pathways, adequate infrastructure, and supportive regulatory frameworks [5].

This mapping also indirectly points to broader gaps in workforce planning and formal recognition of pediatric allergology in Italy. An additional relevant aspect concerns the availability of full-time pediatric allergists within the mapped structures. According to the information collected, the number of full-time specialists varied widely across centers, with larger academic and IRCCS institutions generally hosting a greater number of dedicated allergists compared with smaller territorial structures. This variability may reflect differences in institutional resources and referral patterns and may partly contribute to the heterogeneity observed in weekly clinical activity. Centers with a higher number of dedicated specialists are also more likely to support comprehensive diagnostic pathways, multidisciplinary care, and research activities. In the absence of national data, strategic planning for service development and training opportunities has historically been limited. At an international level, the World Allergy Organization (WAO) highlighted the global shortage of certified allergists and the need for structured training and certification pathways, particularly in countries where subspecialty recognition is fragmented or absent [7]. Similar concerns have been reported in Europe, with surveys highlighting variability and gaps in allergy training provision, including among general practitioners and for systemic allergic disorders [8]. In the United States, the American Academy of Allergy, Asthma & Immunology (AAAAI) Workforce Committee raised concerns regarding the mismatch between rising demand and a potentially diminishing workforce due to aging specialists and limited training positions [9]. Together, these observations reinforce the need for targeted national strategies aimed at reducing geographic inequities, strengthening pediatric allergology training pathways, and supporting multidisciplinary collaboration.

In this context, Daniels et al. [10] emphasized the importance of Integrated Care Pathways (ICPs) and coordinated multidisciplinary approaches to bridge the “second translational gap,” namely the discrepancy between evidence-based recommendations and their consistent application in real-world clinical practice. Their framework is highly relevant to pediatric allergic diseases, which require continuity of care across hospital-based services, community pediatrics, and territorial structures. Drawing on both national [5] and international evidence [7–10], a coordinated approach involving scientific societies such as SIAIP, healthcare institutions, and policymakers appears essential. Such a strategy should aim to harmonize organizational models, promote equity of access through regional networks, support accredited training opportunities, and facilitate the safe implementation of telemedicine and other digital solutions to strengthen continuity of care across Italy.

### Limitations and future perspectives

This study presents several limitations that should be acknowledged. First, the mapping relied on data collected directly by regional SIAIP representatives rather than through a standardized survey instrument. Although this method ensured broad geographic coverage, the heterogeneity in data collection procedures may have introduced variability in reporting accuracy and completeness. Second, the study captured only structures affiliated with pediatric allergology and immunology networks or known to regional representatives, potentially overlooking additional territorial services or private practices not formally linked to SIAIP. Third, the analysis did not assess patient volumes, case severity, staffing levels, or resource availability, which would have provided deeper insight into the functional capacity of each structure. Moreover, the analysis was descriptive and did not adjust the number of structures according to regional population size or pediatric population density. Therefore, the results should be interpreted as an overview of the distribution of services rather than a precise estimate of service availability relative to population needs. Fourth, the classification of structures was based on self-reported organizational models, which may differ across institutions and lack uniform national definitions. Finally, given the absence of previous national data for comparison, temporal trends and changes in service provision could not be evaluated. Despite these limitations, this study represents the first effort to map pediatric allergy and immunology services in Italy and establishes a foundation for future work. Prospective initiatives should prioritize the adoption of standardized data-collection tools, including validated indicators for clinical activity, staffing, diagnostic capacity, waiting times, and patient outcomes. National benchmarking and longitudinal monitoring would allow assessment of service development over time and across regions. Furthermore, future research should explore workforce needs, training gaps, and barriers to access. The expansion of telemedicine, the implementation of integrated care pathways, and the creation of minimum structural and operational standards for pediatric allergy centers represent key opportunities to enhance equity and quality of care nationwide.

## Conclusions

This survey provides the first comprehensive overview of pediatric services in Italy, highlighting the marked heterogeneity in structural organization, service distribution, and resource allocation. Despite the growing demand, many structures operate with limited staff and infrastructure, and significant disparities persist across regions. While there are encouraging examples of excellence, the overall picture reflects an urgent need for national coordination, structured training programs, and formal recognition of this subspecialty. Strengthening workforce capacity and standardizing care pathways are essential to ensure equitable and high-quality allergy care for all children.

## Supplementary Information

Below is the link to the electronic supplementary material.

Supplementary Material 1

## Data Availability

The dataset generated and analyzed during the current study is available from the corresponding author on reasonable request.
