# Supplementary material for: Mapping of pediatric allergy structures in Italy: a nationwide survey
Source: Ital J Pediatr. 2026 Apr 22;52:113. doi: 10.1186/s13052-026-02261-2 (PMC13352835; doi:10.1186/s13052-026-02261-2)
Supplement: Supplementary file 1 — Supplementary Material 1 [file 13052_2026_2261_MOESM1_ESM.docx]

# SIAIP Mapping Project – Survey Questionnaire

This structured questionnaire was developed by the coordinating team of the SIAIP Mapping Project and aims to gather comprehensive data from pediatric allergy and immunology structures in Italy. The questionnaire consists of 16 questions, organized into four thematic sections.

## Section 1: Identification and Location

1. 1. Full name of the structure/clinic
2. 2. City and region
3. 3. Name of the hosting institution
4. 4. Type of structure:

    ☐ Academic Center ☐ IRCCS ☐ Hospital/Community Center
5. 5. Website (if available)

## Section 2: Organizational and Structural Characteristics

1. 6. Name of the medical director or responsible physician
2. 7. Institutional email of the director/responsible physician
3. 8. Number of full-time pediatric allergists/immunologists

## Section 3: Contact and Access Information

1. 9. Phone number(s) for booking appointments
2. 10. Booking hours and working days
3. 11. Modes of access (tick all that apply):

    ☐ Direct booking ☐ Referral only ☐ Urgent/emergency access

## Section 4: Affiliations and Accreditation

1. 12. Is your structure currently accredited by or affiliated with SIAIP activities?
    ☐ Yes ☐ No
2. 13. Does your structure participate in any regional or national care/research networks?
    ☐ Yes ☐ No
3. 14. Would your structure be willing to participate in future national audits or research projects?
    ☐ Yes ☐ No

## Notes

________________________________________________________________________
________________________________________________________________________
________________________________________________________________________
